# High baseline PD-1+ CD8 T Cells and TIGIT+ CD8 T Cells in circulation associated with response to PD-1 blockade in patients with non-small cell lung cancer

**DOI:** 10.1007/s00262-025-04086-0

**Published:** 2025-09-13

**Authors:** Nikita Dutta, Johanna Svensson, George-Alehandro Saad, Marielle Mello, Ella A. Eklund, Ilayda Altinönder, Per Torstensson, Volkan I. Sayin, Anna Rohlin, Hervé Luche, Andreas Hallqvist, Sukanya Raghavan

**Affiliations:** 1 Department of Microbiology and Immunology, Institute for Biomedicine, University of Gothenburg, Sahlgrenska Academy, Gothenburg, Sweden; 2 Department of Clinical Genetics and Genomics, Sahlgrenska University Hospital, Gothenburg, Sweden; 3 Centre d’Immunophénomique-CIPHE (PHENOMIN), Aix Marseille Université, National Institute of Health and Medical Research (INSERM), The French National Centre for Scientific Research (CNRS), Marseille, France; 4 Department of Laboratory Medicine, Institute for Biomedicine, University of Gothenburg, Sahlgrenska Academy, Gothenburg, Sweden; 5 Department of Surgery, Institute for Clinical Sciences, Gothenburg, Sweden; 6 Wallenberg Centre for Molecular and Translational Medicine, University of Gothenburg, Gothenburg, Sweden; 7 Department of Pulmonary Medicine, Skaraborg Hospital, Skövde, Sweden; 8 Department of Oncology, Institute for Clinical Sciences, University of Gothenburg, Sahlgrenska Academy, Gothenburg, Sweden; 9 Department of Clinical Immunology and Transfusion Medicine, Sahlgrenska University Hospital, Gothenburg, Sweden

**Keywords:** Immune monitoring, Lung cancer, Immune checkpoint blockade, CD8 T cells, Circulating tumor DNA

## Abstract

Blockade of PD-1 or its ligand PD-L1 with antibodies revolutionized treatment for stage III and IV non-small cell lung cancer (NSCLC) since FDA approval in 2015. However, resistance to PD-1/PD-L1 blockade remains a challenge, highlighting the need for biomarkers. This study analyzed 36 stage III and IV NSCLC patients, classified as responders or non-responders by iRECIST criteria. Peripheral blood mononuclear cells collected at baseline and post-treatment were examined for surface and intracellular markers via flow cytometry. CITE sequencing of CD8 T cells from three patients and plasma ctDNA analysis from 13 patients was performed using an ultrasensitive barcoding and next-generation sequencing method. Phenotypic analysis of CD8 T cells revealed higher TIGIT and PD-1 expression at baseline in responders compared to non-responders. Long-term responders (> 21 months) exhibited increased TCF-1^+^PD-1^+^ CD8 T cell frequencies relative to shorter-term responders (> 15 months) and non-responders. CITE sequencing revealed intrinsic differences in immune regulation pathways between responders and non-responders. Finally, non-responders showed elevated and increasing ctDNA levels post-treatment, correlating with declining TCF-1^+^PD-1^+^ CD8 T cells. Our data suggests combining CD8 T cell analysis with ctDNA dynamics could identify promising biomarkers for monitoring clinical response and treatment efficacy to PD-1/PD-L1 blockade in NSCLC.

**Supplementary Information:**

The online version contains supplementary material available at 10.1007/s00262-025-04086-0.

## Introduction

The current standard therapy for patients after diagnosis with late-stage non-small cell lung cancer (NSCLC) is treatment with programmed death receptor protien-1 (PD-1), or PD-L1 blockade alone or in combination with chemotherapy. However, with response rates of 30–45%, it is still not fully understood why some patients respond to PD-1/PD-L1 blockade while other patients can completely fail to respond [1]. Biomarkers for treatment response in patients with NSCLC patients are crucial. The current study aims to identify distinct phenotypic and transcriptomic characteristics of circulating CD8 T cells in groups of patients responding to therapy compared with a group of non-responders, potentially yielding novel indicators of treatment efficacy or disease progression. Phenotypic analyses of CD8 T cell subsets can be challenging and tracking antigen-specific T cells is difficult due to their low frequency in the blood. To improve the predictability of biomarkers of clinical response, one strategy can be to combine phenotypic with genetic analyses [2].

PD-1 has been used as an activation marker during antigen recognition. However, it is also expressed along with other inhibitory receptors such as TIGIT, LAG-3, and TIM-3 during chronic stimulation [3] Previous studies suggest that peripheral CD8 T cells may serve as biomarkers of response to PD-1 blockade, with one study linking PD-1^+^ CD8 T cell expansion to clinical benefit and another associating reduced PD-1 expression after one treatment cycle with durable responses [3, 4]. Both T cells and natural killer (NK) cells significantly upregulate TIGIT upon activation [5]. TIGIT binds to CD155, which is expressed on antigen-presenting cells or tumor cells, to suppress the functions of T cells. TIGIT as a critical immune checkpoint, capable of impairing multiple stages of the cancer immunity cycle and inhibiting effective anti-tumor responses [5]. TIGIT expression on tumor-infiltrating lymphocytes (TILs) showed a significant correlation with PD-1 expression and was strongly associated with negative prognostic factors in melanoma [6].

CD8 T cells in the context of persistent antigenic stimulation can become dysfunctional or exhausted, where they can lose their ability to effectively combat tumors [7]. T cell exhaustion stems from prolonged T cell activation and transcriptionally, activated CD8 T cells share similarities with exhausted CD8 T cells, reflecting the role of chronic activation in driving T cell exhaustion [8]. It is difficult to dissect the differences between activation and exhaustion solely from phenotypic markers due to an overlap in surface markers between activated CD8 T cells and exhausted CD8 T cells. However, exhausted T cells in the tumor microenvironment exhibit distinct hallmarks, including loss of proliferative capacity, loss of effector function, and a unique transcriptional profile compared to effector or memory T cells, known to rapidly respond to antigens [9].

The β-catenin/TCF-1, T cell differentiation T cell factor signaling pathway regulates T cell differentiation both in the thymus and in peripheral lymphoid tissues. β-catenin/TCF signaling is essential for maintaining hematopoietic stem cell self-renewal and promoting the proliferation of progenitor cells [10]. Recent studies have highlighted the importance of TCF-1⁺PD-1⁺ CD8 T cells also referred to as stem-like or progenitor exhausted CD8 T cells, in orchestrating effective T cell responses [11, 12]. Miller et al. identified three distinct subsets of exhausted T cells—progenitor, intermediate, and terminally exhausted—each with unique functional characteristics. Progenitor exhausted T cells, characterized by expression of TCF-1 and exhaustion markers, possess self-renewal capacity and enhanced proliferation post PD-1/PD-L1 blockade. The TCF-1^+^PD-1^+^ CD8 T cells, also known as stem-like or progenitor exhausted CD8 T cells, play a crucial role in T cell responses [13]. In contrast, terminally exhausted, CD8 T cells can be distinguished by high expression of TOX, PD-1, Eomes, and Tbet. Terminally exhausted, CD8 T cells instead lack self-renewal capacity and cannot be reinvigorated by PD-1/PDL-1 blockade. [14].

Integrating phenotypic and molecular signatures of CD8 T cells could better distinguish responders from non-responders to PD-1 blockade [2]. To test this hypothesis, we employ cellular indexing of transcriptomes and epitopes by sequencing (CITE-seq), a technique that simultaneously profiles surface proteins (phenotypic markers) and RNA transcripts (genetic profiles) at the single-cell level [15]. This approach enables us to comprehensively characterize circulating CD8 T cells, identifying molecular signatures that may predict treatment outcomes.

While immune cell profiling provides insights into response mechanisms to PD-1/PD-L1 blockade, circulating tumor-derived DNA (ctDNA) offers a complementary approach, reflecting the tumor burden at the time of sampling. Exploring the interplay between immune cells during treatment and ctDNA levels could yield novel predictive tools for clinical response. Indeed, in patients with advanced NSCLC undergoing PD-1/PD-L1 blockade therapy, ctDNA levels in circulation have been shown to indicate survival outcome [16, 17]. Although ctDNA levels reflect on the effect of immunotherapy on the tumor burden their association to the immune cells in circulation has not been explored [18]. This study seeks to monitor the ctDNA levels, and changes in circulating immune cell subsets, potentially uncovering a link between tumor dynamics and immune responses.

Our analysis reveals a significant difference in baseline immune profiles, with CD8 T cells from responders exhibiting higher expression of TIGIT and PD-1. Post-treatment, effector memory CD8 T cells in responders were activated and produced cytokines, suggesting immune activity. Notably, TCF-1^+^PD-1^+^ CD8 T cells increased in some long-term responders, a trend not observed in non-responders. Using CITE sequencing, we report that *TCF7* upregulation in CD8 T cells in responders indicates that progenitor exhausted T cells were detectable in circulation before treatment. These findings underscore the potential of baseline immune profiling and post-treatment T cell responses as predictors of PD-1/PD-L1 blockade efficacy in patients with NSCLC.

## Materials and methods

### Patient cohort and study design

**Fig. 1:**
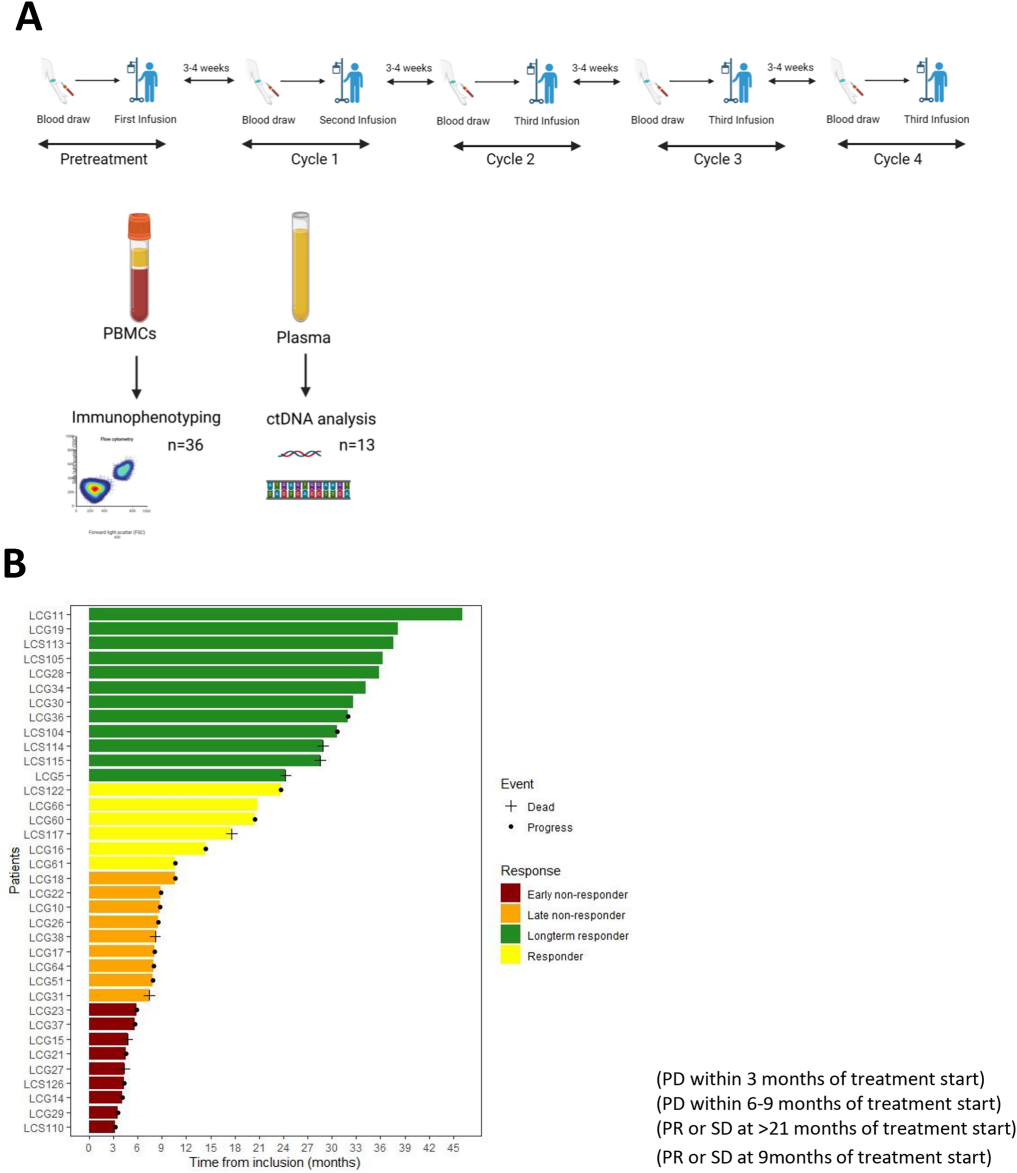
Study Overview. **A** Blood and plasma samples was collected every 3–4-week time points over the treatment period for up to 4 cycles. PBMCs were isolated from the blood and used for flow cytometry analysis and plasma was collected for ctDNA analysis. **B** Swimmer’s plot indicating response to PD-1/PD-L1 blockade in the cohort of patients

This prospective study was comprised of 36 patients diagnosed with stage III or IV adenocarcinoma, squamous cell carcinoma or NOS (not otherwise specified) and recruited between April 2019 and July 2023. Written informed consent was obtained from all patients prior to inclusion (ethical approval number: 953/18). Patients received treatment with antibodies to PD-1 or PD-L1 (PD-1/PD-L1 blockade) as monotherapy (*n* = 17) or in combination with chemotherapy (*n* = 19) as a part of the standard clinical practice (Table 1). Peripheral blood was collected from each patient prior to treatment and post-treatment at each consecutive treatment cycle (2–4 weeks) for up to 5 cycles (Fig. 1A). Treatment cycles of 2–3 weeks were scheduled according to the approved drug interval (Fig. 1A).

**Table 1:** Patient cohort

Patient cohort	n = 36
Median age (range)	76 (65–82)
*Sex, n (%)*	
Male	15
Female	21
*Histology/diagnosis, n (%)*	
Adenocarcinoma	25
Squamous cell carcinoma	11
NSCLC NOS (Not otherwise specified)	0
*PD-L1 status, n (%)*	
1–50%	14
> 50%	17
Negative for PD-L1	3
No material or not determined	2
*Stage at diagnosis, n (%)*	
III	8
IV	26
I	2
*PD-1 Blockade*	
Pembrolizumab	9
Nivolumab	1
Atezolizumab	1
Durvalumab	6
Pembrolizumab + Chemotherapy	19
*Disease response at 9 months, n (%)*	
Partial responder	15
Stable disease	3
Early progression (within 3–6 months)	15
Late progression (within 9 months)	3

### Clinical response evaluation

Clinical responses was evaluated in line with the Immune-related Response Evaluation Criteria for Solid Tumors (irRECIST) and reviewed by an oncologist based on clinical judgment based on CTScan reports [19] Clinical response at 9 months cut-off was defined as complete response (CR, no measurable tumor), partial response (PR, shrinkage in tumor size compared to baseline), stable disease (SD, no change in tumor size compared with baseline), or progressive disease (PD, increase in tumor size compared with baseline). Responders were defined as patients with either (1) complete response, (2) partial disease, or (3) stable disease 9 months after therapy start. Responders longer than 21 months were categorized as long-term responders. Nonresponders at 3 months were defined as early progression and at 6 or 9 months defined as late progression.

### Peripheral blood mononuclear cells (PBMCs) isolation

Blood samples were stored overnight at room temperature before isolation of peripheral blood mononuclear cells (PBMCs) by density gradient centrifugation using Ficoll-Paque™ PLUS (GE Healthcare) followed by cryopreservation in fetal calf serum (FCS) (Sigma) and 10% dimethyl sulfoxide (Sigma/Merck). PBMCs placed in cool boxes at −70 °C before transfer to liquid nitrogen tanks for long-term storage.

### Plasma collection and ctDNA extraction and analysis

Blood was stored at room temperature overnight before isolation of plasma stored at −70 ^°^C. Before ctDNA extraction, plasma was centrifuged at 16,000 g for 10 min at 4 °C. Cell-free DNA was extracted using EZ1&2 Qiagen ccfDNA Kit as per manufacturer’s instructions (Qiagen). CtDNA was quantified using Simsen® Personal (Simsen Diagnostics, Sweden). Based on Simsen-Seq, targeted amplicon sequencing panels was developed for each patient [20]. The panels were developed from the patient’s tumor mutational profile where 4–14 patient-specific variants were used. The designed panels was then applied to monitor ctDNA content in the plasma at up to 6 consecutive timepoints. Sequencing was performed on an Illumina NovaSeq instrument (San Deigo, CA, USA). Bioinformatics and data analysis were conducted using unique molecular identifiers (described in PMID: 36,031,761). The change in ctDNA levels was analyzed for 5 consecutive time points (Fig. 1A) for each patient by assessing the variant allele frequency (VAF) of a selected number of variants (~ 4–14) represented in the tumor of the same patient. A ctDNA positive sample was defined as having at least 2 mutant molecules (MM) per ml plasma in total, summarized over all assays. Whereas non-detectable (ND) levels of ctDNA was defined as having in total 1 MM/ml, or none. For a specific variant to be detected, at least 2 MM/ml should be present in one timepoint. The estimated ctDNA levels was referred to as ctDNA load [20].

### Flow cytometry—acquisition and analysis

PBMCs (0.1 million cells/well) were stained with antibodies against surface markers for 20–30 min at 4 °C (Table 2). Panels for T cell phenotyping were established according to the human standard phenotyping panel [21]. Furthermore, comprehensive panels for both effector and regulatory T cells, intracellular cytokine expression, activation and co-stimulatory and immune checkpoint receptor expression were established. Intracellular staining with antibodies was performed with the buffers from the FoxP3 staining kit (eBioscience). Samples were acquired on a LSRFortessaTM X-20 cell analyzer (BD Biosciences). The PD-1 staining in samples post-treatment from patients treated with anti-PD-1/PD-L1, was assessed using a method described by Kamphorst et al. [4]. Briefly, a two-step staining was performed first with anti-human IgG4 (BV605 or PE-Cy7) and after a wash, staining with directly conjugated anti-PD1 (BV605 or PE-Cy7) (Supplementary Fig. 2D). Data analysis was conducted using FlowJo software v10 (Treestar Inc.)

**Table 2:** Flow cytometry antibodies

Fluorochrome	Marker	Clone
BV421	CXCR5	RF8B2
BV605	NKG2D	1D11
BV711	Ki67	B56
PE-Cy7	CD279 (PD-1)	EH12.1
BV421	CD57	NK-1
BV605	NKG2D	1D11
BV711	CD16	3G8
PE-CF594	CD56	NCAM16.2
AF647	Granzyme B	GB11
BV421	CTLA4	BNI3
BV650	CD127	HIL-7R-M21
BV711	Ki67	B56
PE	CD197 (CCR7)	2-L1-A
PE-CF594	FoxP3	236A/E7
PE-Cy7	CD25	M-A251
AF647	HELIOS	22F6
BV650	TIGIT	741,182
PE	TCF1	S33-966
PE-CF594	TIM3	7D3
BV786	T-bet	O4-46
BV605	CD279 (PD-1)	EH12.1
PE-Cy7	CD45RA	HI100
BV711	CD197 (CCR7)	2-L1-A
BUV737	CD137 (41BB)	4B4-1
BV421	ICOS/CD278	C398.4A
PE-CF594	CD134 (OX40)	ACT35
PE-Cy7	CD28	CD28.2
APC	CD40L/CD154	5C3
BUV395	CD3	UCHT1
BUV737	CD45RO	UCHL1
Bv510	Dump/Dead	FVS510
BV786	CD45RA	HI100
FITC	IFNγ	25,723.11
PerCP Cy 5.5	TNFα	MAb11
BB515	HLA-DR	G46-6
BB700	CD38	HIT2
PE	TCF1	S33-966
PE-CF594	TIM3	7D3
AF700	CD4	SK3
APC-Cy7	CD8	SK1

### Single-cell RNA-Seq, cellular indexing of transcriptomes and epitopes by sequencing (CITE-Seq)

PBMCs thawed in HBSS with 2% FCS and SIMP, were incubated with HBSS containing 2% FCS and nuclease. After washing, cells were counted using an Attune NxT flow cytometer (Invitrogen). Cells were then incubated with anti-PD-1 (Keytruda), CD8-PE, CD45RO, and CD4-BV711 for 20 min. CD8^+^ cells were isolated using Dynabeads. For CITE-seq, cells were labeled with barcoded hashtag antibodies on a Curiox laminar plate, followed by 9 washes. Cells were then incubated with an ADT mix for surface protein detection. After pooling and sorting, cells were encapsulated on chip G. cDNA libraries were prepared, followed by gene expression, Antibody-Derived Tags (ADT), and (Hashtag Oligo) (HTO) library generation.

### Sequencing and data analysis

Sequencing was performed by Macrogen (Seoul, South Korea) on an Illumina NovaSeq platform. All bioinformatics analyses were performed on a local server running Ubuntu 22.04, equipped with 40 CPU cores and a total 128 GB of RAM. The server hosted an R Studio service, using R version 4.2.2 and RStudio version 2022.02.1 + 461. mRNA libraries were preprocessed using CellRanger count (v. 7.1.0). The alignment was performed against the human genome reference refdata-gex-GRCh38-2020-A.

For counting ADT and HTO data, CITE-seq-Count (v1.4.5) was used. Each of the 6 pooled sample was demultiplexed using the HTODemux function with the default parameters of the Seurat package (v.4.1.0): cells retained as singlets were those whose HTO signal exceeds the 99th percentile for one hashtag. So only cells with successful patient identification were retained for further analysis (81.8% of the aligned cells).

Quality control steps were performed to eliminate low-quality cells corresponding to cells expressing less than 200 genes or cells > 15% of mitochondrial-associated genes. Genes expressed in less than three cells were also removed. After these filtering steps, 74.09% of the aligned cells are included in the following analyses. Raw expression counts were normalized and scaled using, respectively, the Seurat NormalizeData and ScaleData functions. The first 2000 variable genes were selected using Seurat's FindVariableFeatures function. The first 30 principal components (PCs) were calculated. UMAP was performed using these calculated PCs. To find the differences and similarities between non-responder and responder patients, data have been integrated using FindAnchors and Integrate functions of Seurat package. The 14 clusters were identified using the Seurat FindClusters function, which applies the Louvain algorithm on a shared nearest neighbor (SNN) graph constructed from the calculated PCs, with clustering resolution set to 0.48.

### Statistics

Non-parametric Mann–Whitney *U*-tests or Wilcoxon matched-pairs signed rank test was performed for unpaired and paired analyses respectively using the GraphPad Prism software (v7.0). Statistical significance was set at *p* < 0.05.

## Results

### Patient characteristics

This prospective study of NSCLC patients receiving PD-1/PD-L1 blockade included 36 patients, divided equally between responders and nonresponders. Among responders, 11 achieved long-term response (> 21 months), while the rest of the patients relapsed between 9 and 15 months. CITE-seq analysis was performed on PBMCs isolated from two long-term responders and one early non-responder (3 months). A swimmer’s plot illustrates the response pattern for the patient cohort (Fig. 1B). A summary of the patients’ characteristics is described in Table 1.

### Expression of TIGIT/PD-1 and TCF-1^+^PD-1^+^ CD8 T cells could predict response to PD-1/PD-L1 blockade in NSCLC

To investigate baseline patient characteristics and monitor changes over time during treatment with PD-1/PD-L1 blockade, we analyzed CD8 and CD4 T cells in patient blood using flow cytometry with the in-house designed panels. The extensive panels included memory, activated, cytokine secreting T cells, and T cells expressing checkpoint inhibitors or co-stimulatory molecules (Table 2).

**Fig. 2:**
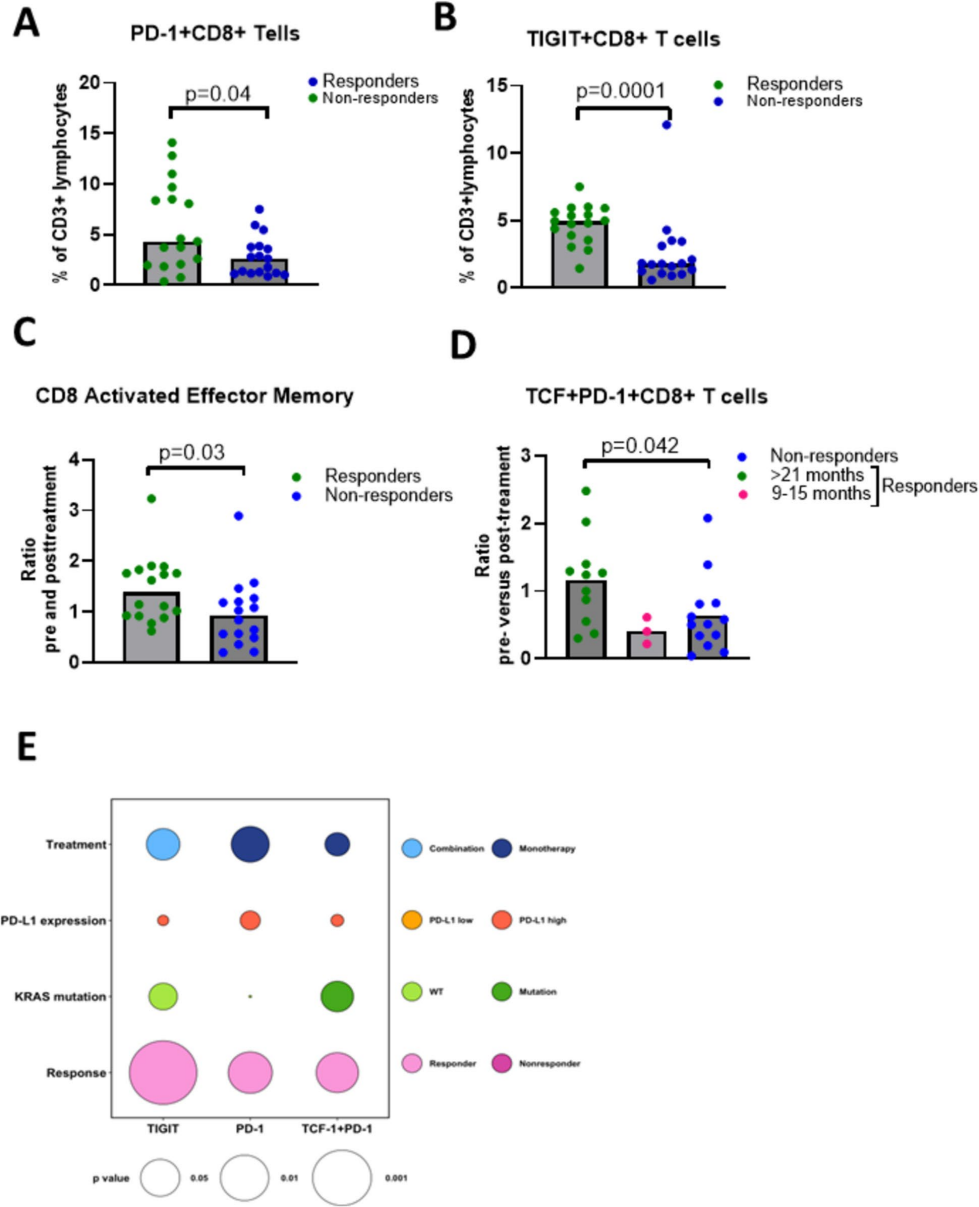
Enhanced baseline expression of PD-1 and TIGIT on CD8 T cells and post-treatment TCF-1^+^PD-1^+^ T Cells in responders to PD-1/PD-L1 blockade. **A** Expression of PD-1 on CD8 T cells and **B** TIGIT on CD8 T cells as a percentage of CD3^+^ lymphocytes at baseline in responders and non-responders. **C** Ratio pre- versus post-treatment activated effector memory CD8 T cells (CD45RA-CCR7-CD38^+^HLA-DR^+^). **D** Expression of TCF-1 and PD-1 on CD8 T cells. Patients were classified with a response of > 21 months, 9–15 months and non-responder at 9 months. **E** Bubble plot analysis of baseline frequencies of PD-1^+^, TIGIT^+^ on CD8 T cells and ratio of post to pre-treatment of TCF-1^+^PD-1.^+^ CD8 T cells among the responders and nonresponders. Non-parametric Mann–Whitney U-tests for unpaired data sets was performed. Statistical significance was set at *p < 0.05. **p < 0.01, ***p < 0.001

In an analysis of stage III/IV patients with NSCLC, comparing 18 responders and 18 non-responders, we found in our cohort of patients that CD8 T cells from responders showed significantly higher PD-1 expression at baseline compared to nonresponders (*p* = 0.04, Fig. 2A). In addition, a significantly higher expression of TIGIT on CD8 T cells, was found in responders compared to nonresponders at baseline (*p* < 0.0001, Fig. 2B and (Supplementary Fig.  2 A). In contrast, TIM-3 expression did not differ between the responders and nonresponders (Supplementary Fig.  2 B). The overall frequencies of CD3, CD8, and CD4 T cells did not vary between responders and non-responders, or during the treatment (Supplementary Fig.  1 A) [22]. High expression of PD-1 and TIGIT on CD8^+^ T cells from responders could indicate that the cells were tumor specific and that T cells were activated. The presence of tumor specific CD8 T cells in blood is known to be beneficial for response to PD-1/PD-L1 blockade [23].

We next included both baseline analysis, and changes in immune cell subsets before and after treatment. Notably, the changes in frequencies of immune cell subsets analyzed peaked following the first treatment cycle (*data not shown*) compared to baseline. Therefore, data presented are comparisons of baseline and after the first treatment cycle. Among the CD8 T cells, the activated effector memory T cells (CD45RA-CCR7-CD38^+^HLA-DR^+^) was significantly increased (*p* = 0.03) after the first treatment cycle (Fig. 2C), but not the activated total CD8 or CD4 T cells (CD4^+^/CD8^+^CD38^+^HLA-DR^+^) (Supplementary Fig. 1B). To understand the functional potential of the CD8 T cells and their relation to clinical response, we examined cytokine expression of IFNγ and TNFα. Our analysis revealed that the baseline TNFα expression by CD8 T cells was similar between response groups. In contrast, the baseline IFNγ expression showed a trend toward being higher in nonresponders compared to responders to PD-1/PD-L1 blockade (Supplementary Fig.  1 C), though the difference was not statistically significant. Notably, within the nonresponder group, patients with higher baseline IFNγ levels were early non-responders. The cytokine expression of IFNγ and TNFα was unaffected by PD-1/PD-L1 blockade, and in responders compared to nonresponders (Supplementary Fig.  3 A and B).

We next proceeded to investigate the presence of progenitor exhausted T cells TCF-1^+^PD-1^+^ CD8 T cells post-treatment as they have been implicated in the response to PD-1/PD-L1 blockade [24]. Our results revealed that in patients with a long-term response (≥ 21 months), there was a significant increase in the ratio of TCF-1^+^PD-1^+^CD8 T cells pre- and post-treatment (*p* = 0.04) which was absent in short term responders (< 15 months) (Fig. 2D). On the contrary, there was a reduction in the frequency of TCF-1^+^PD-1^+^CD8 T cells in nonresponders’ pre- compared to post-treatment (Supplementary Fig.  2 C, D).

**Fig. 3:**
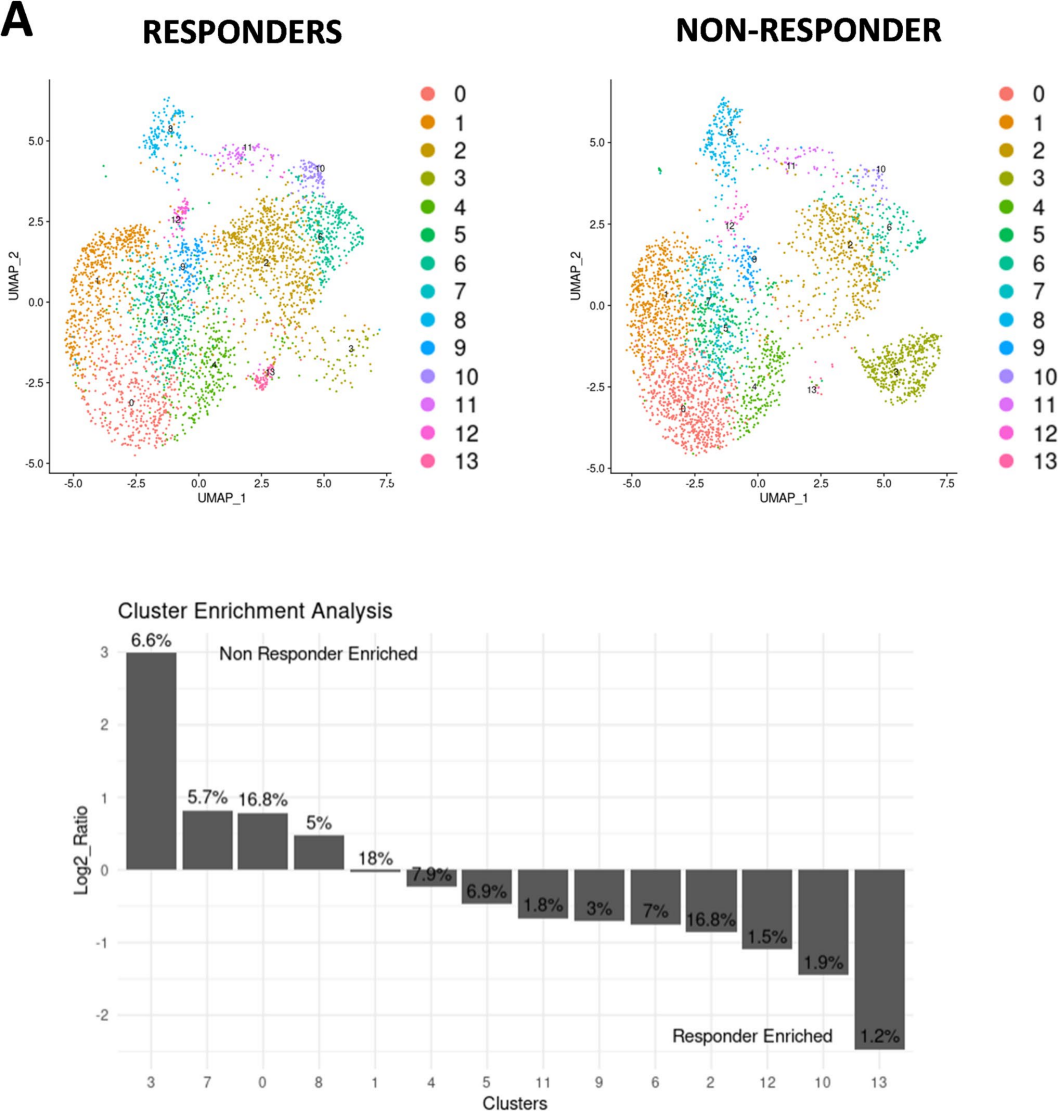

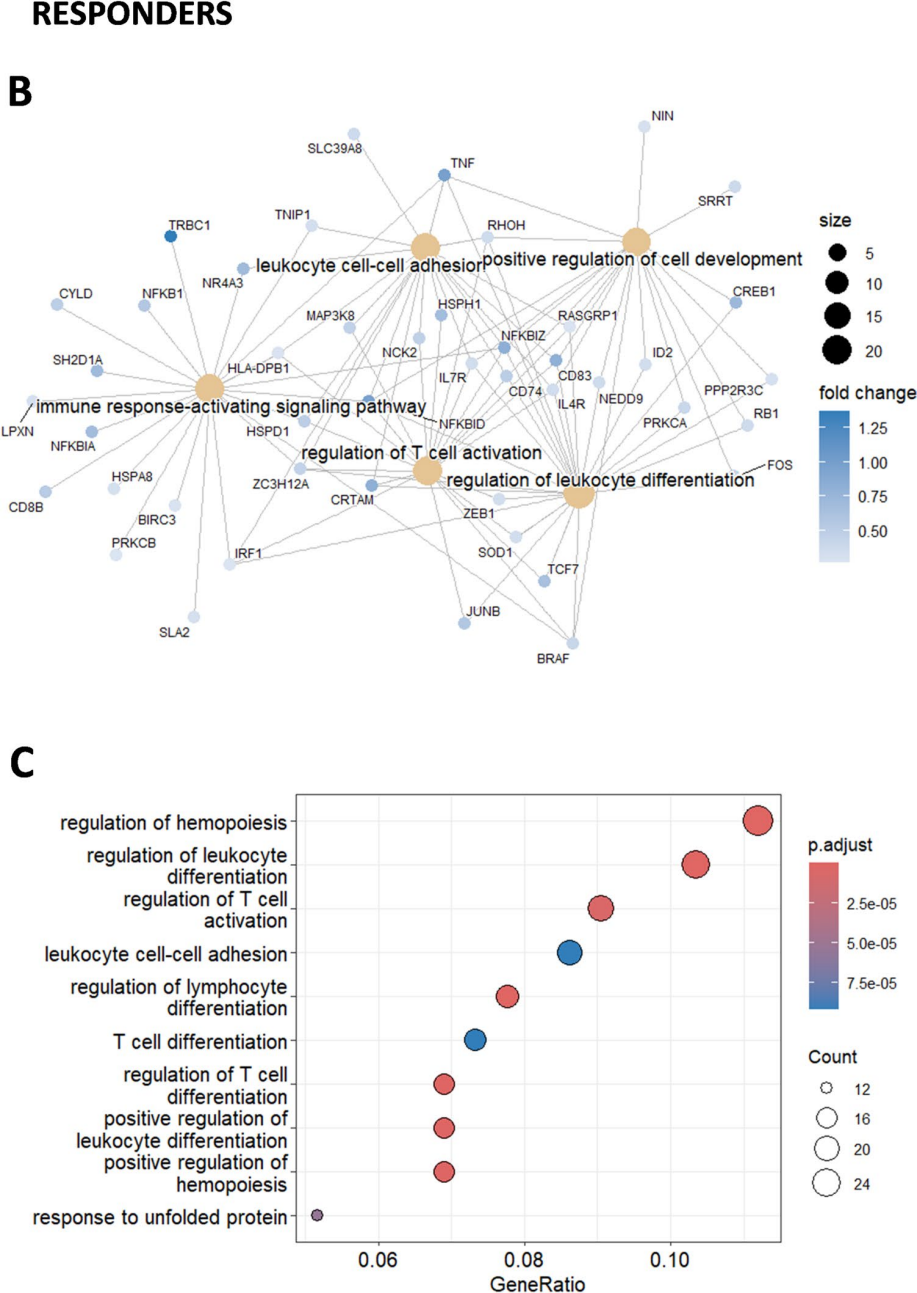

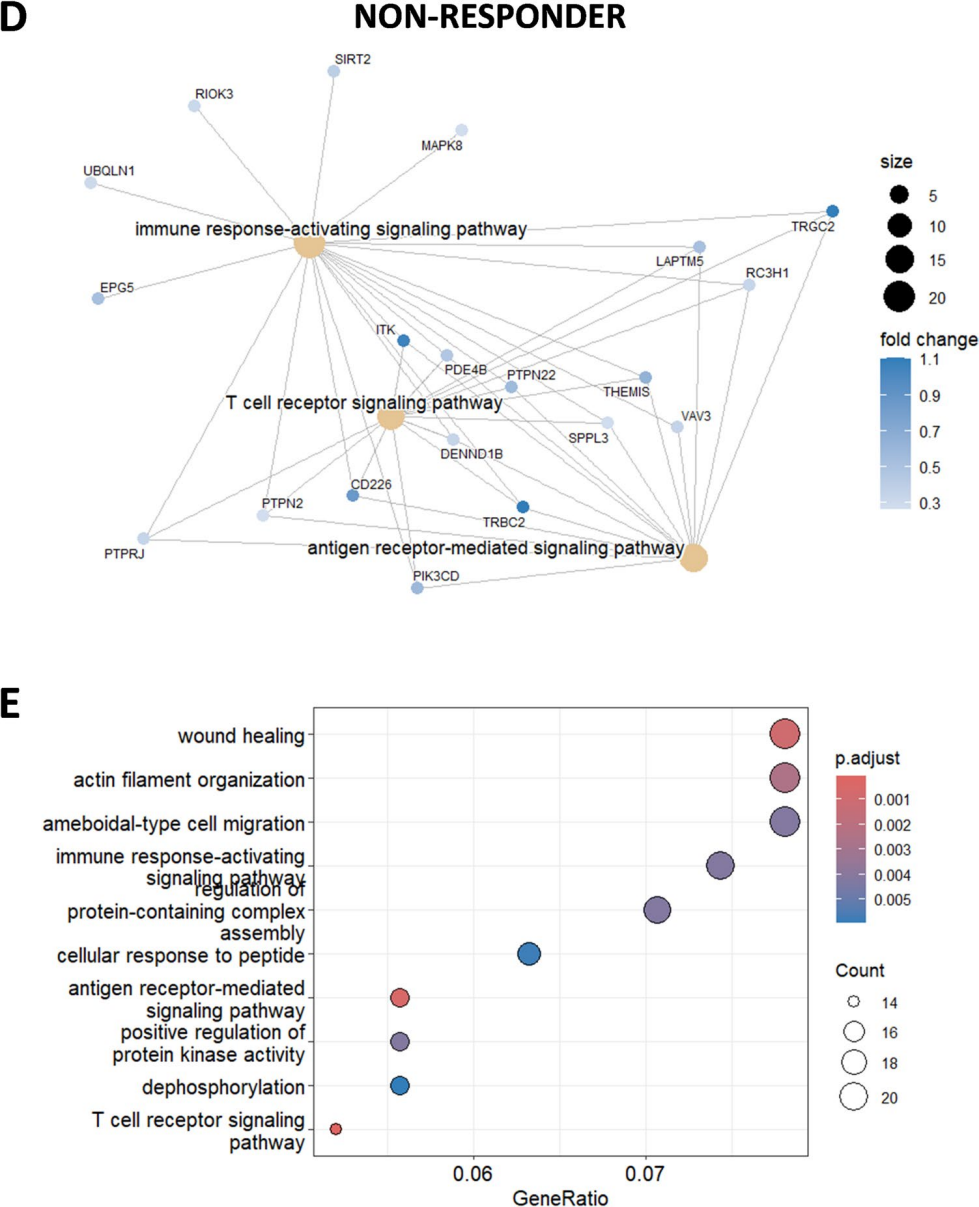
CITE-seq and UMAP analysis reveal baseline CD8 T Cell signatures associated with response to PD-1/PD-L1 blockade **A** CITE sequencing data for simultaneous analysis of surface markers and gene expression. Cluster enrichment analysis show the percent representation of each cluster in the entire population. **B**, **C**, **D** & **E** DGE analysis depicting upregulated genes in both responders and nonresponders. Non-parametric Mann–Whitney U-tests for unpaired data sets was performed. Statistical significance was set at *p < 0.05. **p < 0.01, ***p < 0.001

Analysis of association of clinical parameters with expression of TIGIT and PD-1 on CD8 T cells revealed that the TIGIT expression on CD8 T cells could be significantly associated with response to PD-1/PD-L1 blockade (Fig. 2E). Overall, PD-L1 expression on tumor tissue (tumor proportion score) had no correlation with either TIGIT or PD-1 expression. We also report that KRAS mutation had no correlation with either TIGIT or PD-1 expression or TCF-1^+^PD-1^+^CD8 T cells. Patients in the monotherapy group tended to have high PD-1 expression on CD8 T cells (*p* = 0.06). However, the number of patients in each treatment group, monotherapy (*n* = 17), and combination (*n* = 19) should be noted.

In summary, responders to PD-1/PD-L1 blockade exhibit a significantly higher expression of TIGIT and PD-1 than nonresponders, and long-term responders had an increased presence of TCF-1^+^PD-1^+^CD8^+^ T cells post treatment. These findings suggest that both baseline and post-treatment monitoring of patients could be useful for identification of biomarkers after validation in a larger cohort of patients to predict response to PD-1/PD-L1 blockade.

### CITE-seq and UMAP analysis reveal baseline CD8 T Cell signatures associated with response to PD-1/PD-L1 blockade

To explore molecular signatures of the CD8 T cells and their relation to clinical response, we employed CITE-seq analysis and UMAP clustering of CD8 T cells isolated from two responders and one nonresponder. Analysis of molecular signatures pre- and post-treatment did not reveal any differences between responders and non-responders and therefore results below were focused on the data obtained at baseline. UMAP clustering analysis revealed two CD8 T cell clusters (3 and 13) that distinguished responders from nonresponders at baseline. Cluster 3 represented MAIT cell phenotype, while cluster 13 included mainly memory T cells (Fig. 3A and Supplementary 3 C).

Further, gene expression pattern in single cell CD8 T cells at baseline showed significant upregulation of genes associated with T cell receptor signaling and differentiation. These genes included *TCF7*, *FOS*, *JUN*, *NFKBIZ*, *CD74*, *TNF*, and *CD83*. A upregulation of *TCF7* that encodes for TCF-1 in CD8 T cells was observed among responders compared to nonresponder patient that could suggest a progenitor exhausted CD8 T cell signature (Fig. 3B). Another finding was the upregulation of IL*−4R* in CD8 T cells from responders, which is noteworthy since a recent study highlighted that anti-PD-1 preferably binds to follicular helper T cells which triggers the release of IL-4 that in turn boosts CD8 T cell responses in the draining lymph node and tumor immunity (Fig. 3B). [25] The gene ratio was defined as the ratio between the number of genes of interest associated with a given gene ontology (GO) term and the total number of genes of interest analyzed. The gene ratio cut was made based on the level of unrelated GO terms appearing in the results. Finally, GO analysis revealed a greater gene ratio (> 0.07) in responders compared to nonresponders, in pathways related to hematopoiesis, leukocyte differentiation, and T cell activation, all important for the development of tumor immunity (Fig. 3C).

In contrast, the CD8 T cells from the non-responder patient exhibited minimal upregulation of genes related to immune activation and T cell receptor signaling pathways (Fig. 3D and E) and expressed LAPTM5, a negative regulator of TCR signaling. [26] Altogether, our baseline analysis of gene expression patterns in CD8 T cells from the blood reveals differences in pathways of T cell activation between responders and nonresponders, offering insights into potential mechanisms influencing treatment outcome.

### Analysis of immune cell dynamics and ctDNA load as prognostic markers of response to treatment

**Fig. 4:**
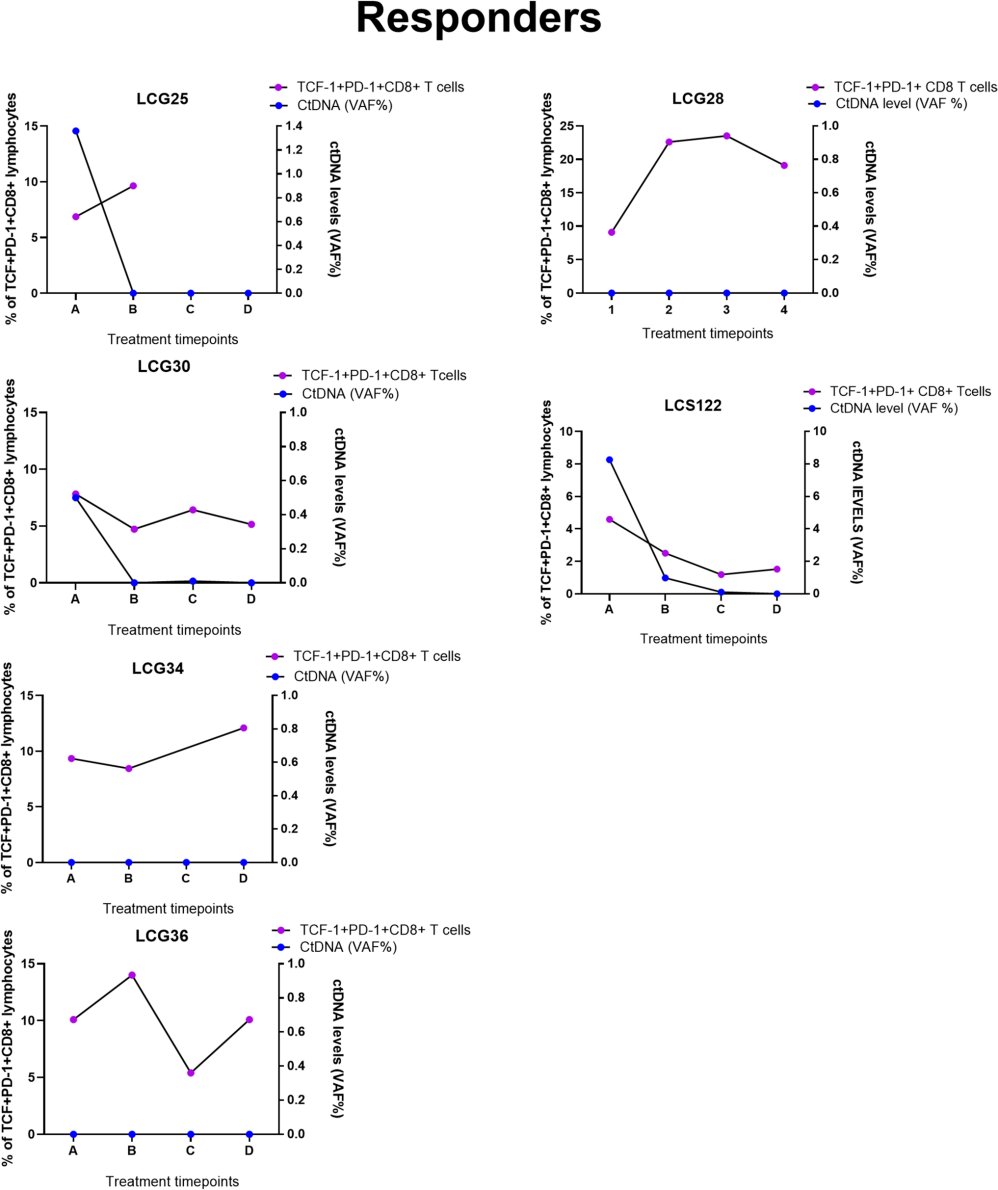

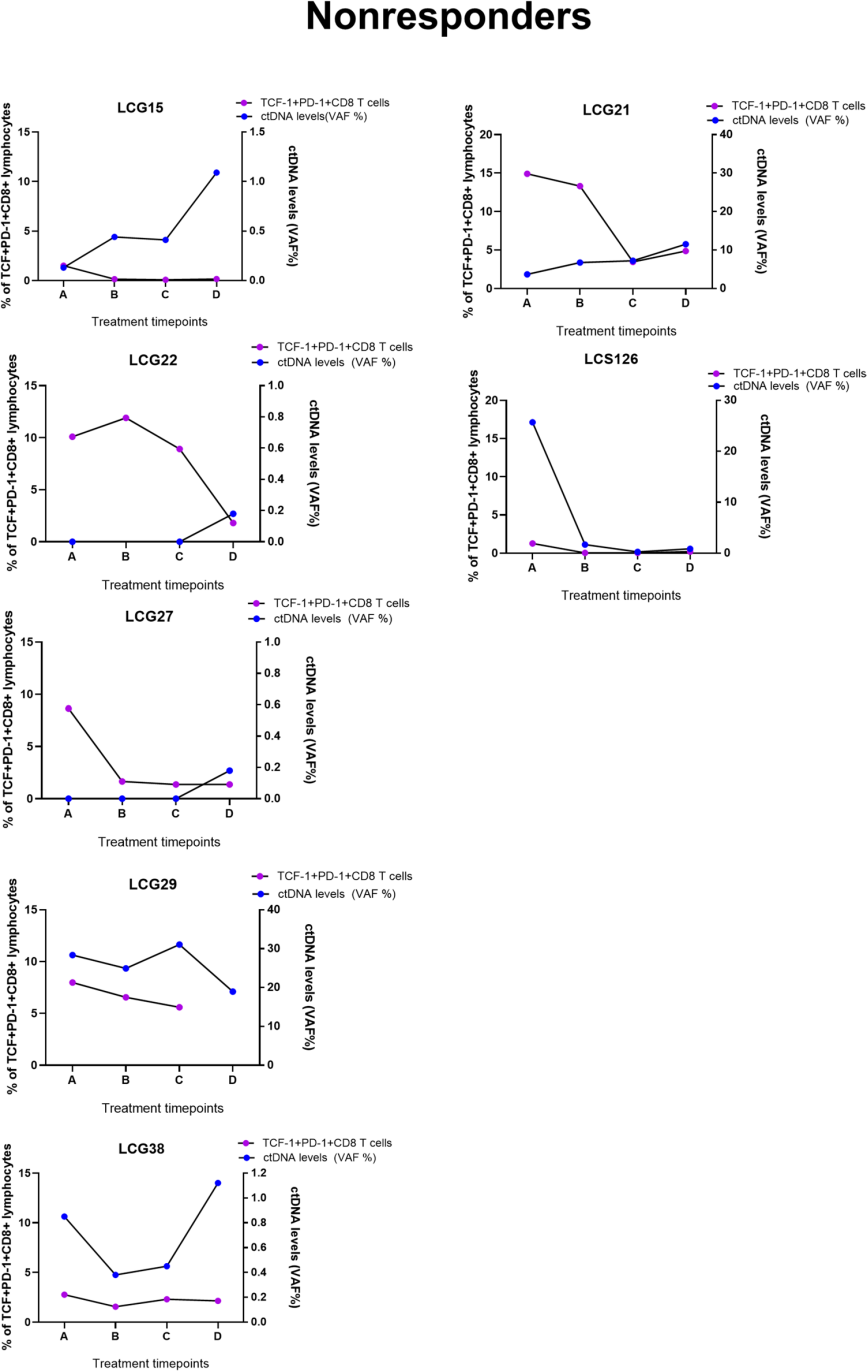
Circulating tumor DNA (ctDNA) analysis in the plasma of responder and non-responder patients’ post-treatment. TCF^+^PD-1^+^ CD8 T cells frequencies post-treatment was plotted against ctDNA levels evaluated from plasma at the same time points for **A** responders and **B** nonresponders

In a subset of the cohort, six responders and seven nonresponders overlapped where both flow cytometry and ctDNA level analysis (including baseline and pre-/post-treatment changes) were included (Fig. 1A). This enabled the investigation of the relationship between circulating immune cells and variations in ctDNA levels over time as a surrogate marker for tumor size post-treatment. In four long-term responder patients**,** TCF-1^+^PD-1^+^CD8 T cells increased over time, while the ctDNA remained undetectable at the same timepoints. However, in patient LCG025, we were able to record ctDNA levels only from two timepoints (Fig. 4A). In 4/7 nonresponders, ctDNA levels remained elevated despite ongoing treatment, while the levels of TCF-1^+^PD-1^+^ CD8 T cells in the blood remained low (Fig. 4B). In three nonresponders, the increase in ctDNA levels in the plasma coincided with declining TCF-1^+^PD-1^+^ CD8 T cells in the blood at synchronous time points. In one nonresponder patient, LCG022, although ctDNA levels remained low and had an initial increase in TCF-1^+^PD-1^+^ CD8 T cells, TCF-1^+^PD-1^+^ CD8 T cells decreased over time. It is important to note the variability in the scale of VAF% among nonresponders (Fig. 4B). Our data suggest that analysis of ctDNA levels coupled with T cell subsets maybe a useful for monitoring response to PD-1/PD-L1 blockade therapy, meriting clinical validation in a larger cohort of patients.

## Discussion

Responders and nonresponders to PD-1 blockade exhibit inherent differences in the CD8 T cells infiltrating the tumor. However, it remains unclear whether such differences can be comprehensively detected in the blood of NSCLC patients as biomarkers of clinical response. Our findings from this study suggest that peripheral blood CD8 T cell responses could reflect clinical outcomes after PD-1 blockade. However, our study also addresses transcriptomic differences in circulating CD8 T cells alongside phenotypic changes, providing key insights into potential functional differences in CD8 T cells from patients with NSCLC who respond to PD-1 blockade. Expanding on our previous findings of predictive changes in immune cell subsets and genetic parameters [22], the current study confirms and extends the observation of a marked post-treatment increase in activated effector memory CD8 T cells among late-stage NSCLC patients responding to PD-1/PD-L1 blockade [22].

We find that baseline CD8 T cells from responders to PD-1/PD-L1 blockade have significantly higher PD-1 and TIGIT expression compared to nonresponders. However, the high expression of PD-1 and TIGIT on CD8 T cells from responders could indicate that the cells were tumor specific and that the T cells were activated. In a study by Chauvin et al. that TIGIT was shown to be upregulated on PBMCs following recent encounter with tumor-antigen in patients with melanoma. Circulating TIGIT⁺CD8⁺ T cells were tumor-specific, with TIGIT⁺PD-1⁺ cells (co-expression) displaying greater activation and differentiation than single-positive cells. [27] TILs that co-expressed both markers suggest that they were more differentiated and dysfunctional [27]. Consistent with this, the expression of multiple inhibitory markers on CD8 T cells may indicate a state of dysfunction. Additionally, co-expression of TIGIT and PD-1 on circulating CD8 T cells has been shown to predict response to PD-1 blockade in patients with melanoma and Merkel cell carcinoma. While the TIGIT⁺ population exhibited a broader range of antigen recognition following PD-1 blockade; the magnitude and number were lower compared to the TIGIT⁺PD-1⁺ double-positive population [28].

We report that high frequencies of CD8^+^PD-1^+^ T cells in baseline biopsies from stage III/IV patients with NSCLC receiving PD-1 blockade was indicative of clinical response and survival. In support of our findings, Thommen et al. have found that the CD8^+^PD-1^+^ T cells subset was found to be tumor reactive and via TcR sequencing found to represent a high clonality, with top 30 clones comprising of almost 90% of the TcR repertoire which indicated that a majority of CD8^+^PD-1^+^ T cell clones were tumor specific [29]. We hypothesize that the high expression of TIGIT or PD-1 on peripheral CD8 T cells at baseline in responders could be an indication that they are tumor specific and therefore indicative of response to PD-1 blockade [30, 31].

In a study of patients with melanoma, TCF-1^+^PD-1^+^CD8 T cells in the blood were found to be antigen specific indicating a functional role for the cell subset [32]. Yost et al. report by bulk TcR sequencing, that clones in the blood were shared by the TILs; and in patients with basal and squamous cell carcinoma, new TILs were recruited from the blood of post treatment [33]. Our own findings reveal that long-term responders (≥ 21 months) had an increase in TCF-1^+^PD-1^+^ CD8 T cells post-treatment compared to short-term responders (< 15 months) and non-responders. All non-responders except one showed a significant decrease in TCF-1^+^PD-1^+^CD8 T cells post-treatment. In mouse tumor models, the tumor-draining lymph node is known to harbor TCF-1^+^PD-1^+^ CD8 T cells. Post-treatment, this subset may become reinvigorated leading to its increased presence in circulation. We hypothesize a reservoir of TCF-1⁺PD-1⁺ CD8 T cells are maintained within the draining lymph nodes and that the TCF-1^+^PD-1^+^ CD8 T cells found in the blood post-treatment are on their way to the tumor [34]. However, additional studies such as TcR sequencing need to be performed to confirm this. Notably, changes in the TCF-1^+^PD-1^+^ CD8 T cells frequencies become detectable as early as 3 week post-treatment, underscoring the critical importance of considering timing for detecting these cells in circulation as a biomarker of clinical response, and for designing effective treatment strategies involving anti-PD-1 therapy.

We observe phenotypic changes on CD8 T cells; however, we were interested to further investigate how the transcriptome of CD8 T cells discerned a responder from a nonresponder to PD-1 blockade. CITE sequencing analysis revealed that CD8 T cells from responders exhibited upregulated gene expression related to T cell activation and differentiation. Notably, the CD8 T cells in responders expressed *TCF7* which codes for TCF-1, which was absent in the nonresponder patient. A separate study exploring single cell RNA sequencing of TILs in NSCLC tumors reported that a proportion of the tumor specific T cell clones can express *TCF7* [35]. Additionally, virus-specific TCF1⁺ CD8⁺ T cells play a central role in chronic infection by sustaining the immune response. They exhibit features of both central memory and exhaustion, retain proliferative capacity, and are essential for the response to checkpoint blockade [36]. Based on studies by Kamphorst et al. and Yost et al. showing that the tumor specific T cells in circulation have features of reinvigoration and less dysfunctional than their tumor counterparts, [33, 37] we hypothesize that expression of *TCF7* by CD8 T cells from responders could indicate tumor-specificity and with the potential to be reinvigorated post PD-1/PD-L1 blockade.

Since peripheral blood from patients is the most ideal non-invasive sample to monitor rapid changes to immune subsets post PD-1/PD-L1 blockade, [37] changes in ctDNA levels can in parallel monitor resistance to treatment before a radiological assessment. In patients with lung cancer or renal cancer, tumor shrinkage was observed at the same time as T cell clones were found to be expanded in the tumor and in the periphery [38]. Additionally, in patients with advanced lung cancer on immune checkpoint blockade, clonal expansion of T cells was found to correlate with a reduction in ctDNA highlighting the potential of ctDNA as a real-time marker for tumor burden and disease progression, while immunological changes would still require immunological assays [18]. We therefore advocate for methods such as flow cytometry to capture T cell subsets in the blood and simultaneous analysis of levels of ctDNA. Indeed, a study by Nabet et al. reports the development of an assay, DIREct-ON a non-invasive and multiparameter assay that assessed the ctDNA at baseline and circulating CD8 T cells. A combination of markers, such as fewer CD8 T cells in circulation and a drop in ctDNA burden could predict response to immune checkpoint blockade in patients with NSCLC after the 1 st cycle of treatment [39]. We observed in our cohort of patients that four out of seven non-responders with a low frequency of TCF-1^+^PD-1^+^CD8 T cells post treatment compared to responders and had high ctDNA levels in the plasma that increased over time. In four responders, while TCF-1^+^PD-1^+^ CD8 T cells increased, a decrease in ctDNA levels was observed. We propose that a combined analysis of ctDNA levels and immune cell subsets in the blood could be useful to monitor clinical response to immune checkpoint blockade. Flow cytometry represents a cost-effective approach that can be readily incorporated into routine clinical workflows using existing infrastructure, without the need for additional expensive equipment. Furthermore, only a minimal volume of whole blood is required to perform both flow cytometry and ctDNA analyses, supporting the feasibility of clinical validation of these potential biomarkers of response owing to their simplicity, accessibility, and low cost.

This study is based on a discovery cohort of patients, and the findings require independent validation to confirm the robustness and generalizability of the findings. A notable limitation was the small number of patients available for paired analyses of circulating T cell subsets and circulating tumor DNA (ctDNA), which may have limited the statistical power to detect associations between immune profiles and molecular alterations.

The major strength of the study was the longitudinal monitoring of individual patients over an extended period (> 2 years), offering valuable insights into dynamic immune and molecular changes. Taken together, the expansion of specific immune subsets such as PD-1^+^CD8 T cells and TIGIT^+^CD8 T cells at baseline and TCF-1^+^PD-1^+^ CD8 T cells during treatment as well as ctDNA dynamics may serve as surrogate biomarkers for clinical response. The findings presented in this study warrant validation in larger patient cohorts, to select robust biomarkers of response to PD-1/PD-L1 blockade**.**

## Supplementary Information

Below is the link to the electronic supplementary material.

Supplementary file1 (TIF 761 KB)** Supplementary figure 1: (A) **Baseline frequencies of % CD3, CD8, and CD4 of total lymphocytes in the blood. **(B) **CD38 and HLA-DR activation marker expression on CD8 and CD4 T cells assessed by flow cytometry. **(C) **PBMCs stimulated with PMA and ionomycin for 4 hours with brefeldin A added in the last 2 hours was analyzed for CD8 T cell expression of IFNγ and TNFα expression by CD8+ T cells pre- and post-treatment.

Supplementary file2 (TIF 1002 KB) **Supplementary figure 2: (A) **Flow cytometry plots of PD-1 cell staining using directly conjugated anti-PD-1 and anti-IgG4 antibodies to detect PD-1 masked by the drug. Flow cytometry plots of TIGIT staining. **(B) **TIM-3 expression at pretreatment on CD8 T cells in responders and non-responders. **(C) **Changes in TCF-1+PD-1+ CD8 T cells pre- and post-treatment. **(D) **Flow cytometry plots of TCF-1+PD-1+ CD8 T cell staining.

Supplementary file3 (TIF 992 KB) **Supplementary figure 3: (A) **Ratio pre- versus post-treatment IFNγ and TNFα expression by CD8+ activated effector memory T cells**. (B) **Flow cytometry plots of IFNγ and TNFα CD8 T cell staining. **(C) **UMAP gene expression analysis at pretreatment of CD8 T cells reveal clusters dominant at baseline differentiating responders from a nonresponder.

## Data Availability

The datasets produced and/or analyzed in this study are accessible from the corresponding author upon reasonable request.
