# Supplementary figures and images for: High baseline PD-1+ CD8 T Cells and TIGIT+ CD8 T Cells in circulation associated with response to PD-1 blockade in patients with non-small cell lung cancer
Source: Cancer Immunol Immunother. 2025 Sep 13;74(10):309. doi: 10.1007/s00262-025-04086-0 (PMC12433424; doi:10.1007/s00262-025-04086-0)

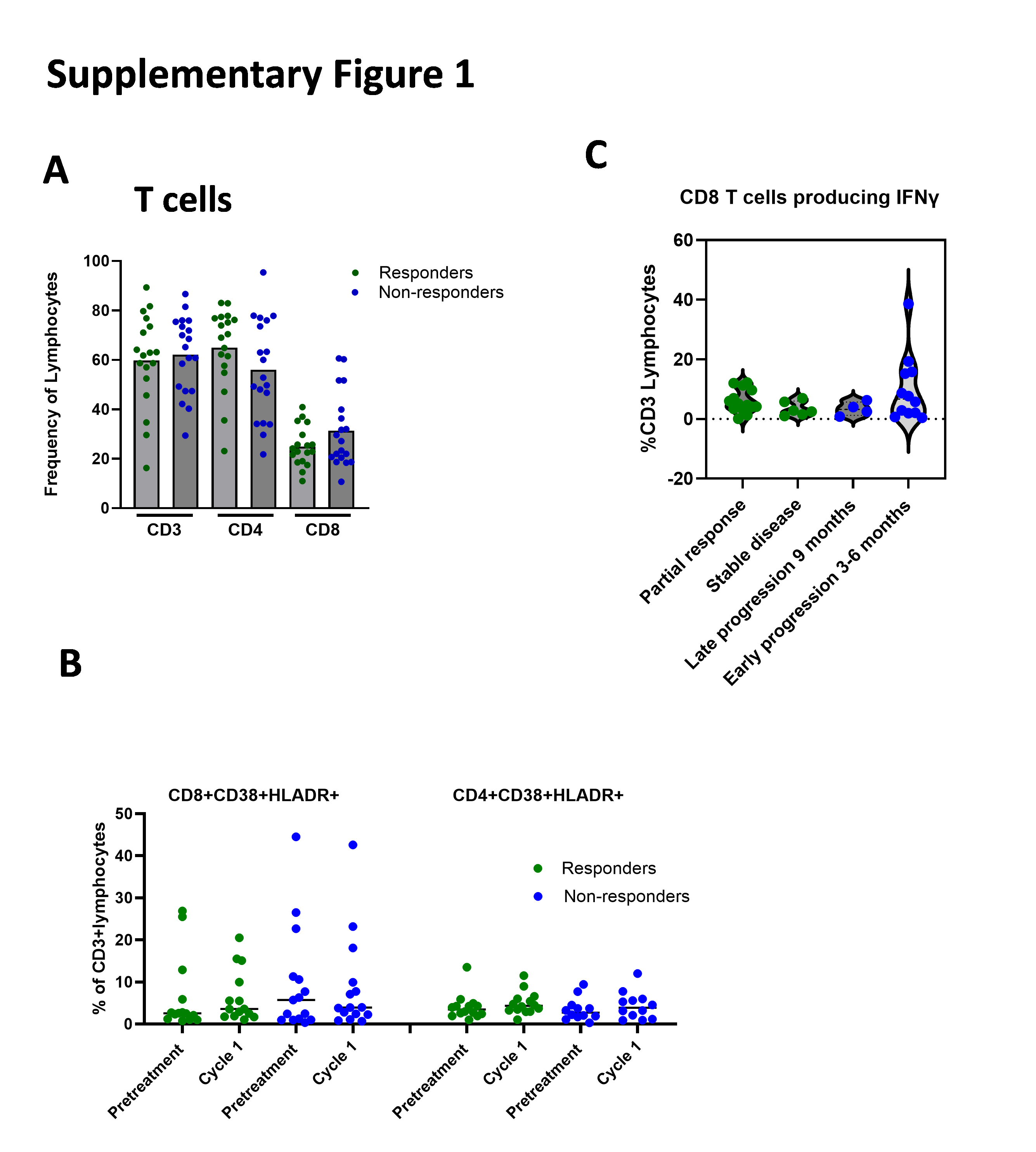

Supplement: Supplementary file 1 — Supplementary file1 (TIF 761 KB) Supplementary figure 1: (A) Baseline frequencies of % CD3, CD8, and CD4 of total lymphocytes in the blood. (B) CD38 and HLA-DR activation marker expression on CD8 and CD4 T cells assessed by flow cytometry. (C) PBMCs stimulated with PMA and ionomycin for 4 hours with brefeldin A added in the last 2 hours was analyzed for CD8 T cell expression of IFNγ and TNFα expression by CD8+ T cells pre- and post-treatment. [file 262_2025_4086_MOESM1_ESM.tif]

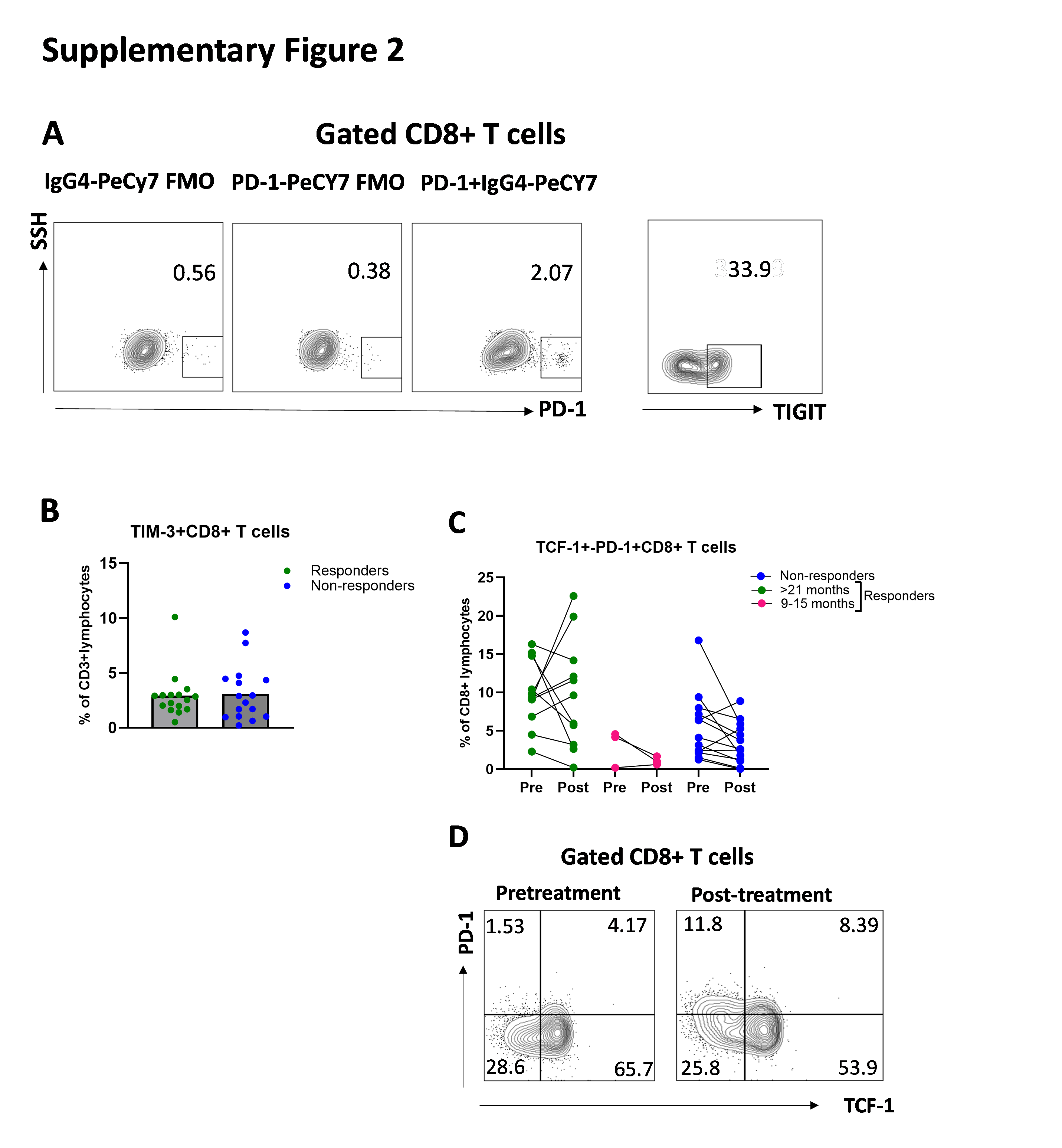

Supplement: Supplementary file 2 — Supplementary file2 (TIF 1002 KB) Supplementary figure 2: (A) Flow cytometry plots of PD-1 cell staining using directly conjugated anti-PD-1 and anti-IgG4 antibodies to detect PD-1 masked by the drug. Flow cytometry plots of TIGIT staining. (B) TIM-3 expression at pretreatment on CD8 T cells in responders and non-responders. (C) Changes in TCF-1+PD-1+ CD8 T cells pre- and post-treatment. (D) Flow cytometry plots of TCF-1+PD-1+ CD8 T cell staining. [file 262_2025_4086_MOESM2_ESM.tif]

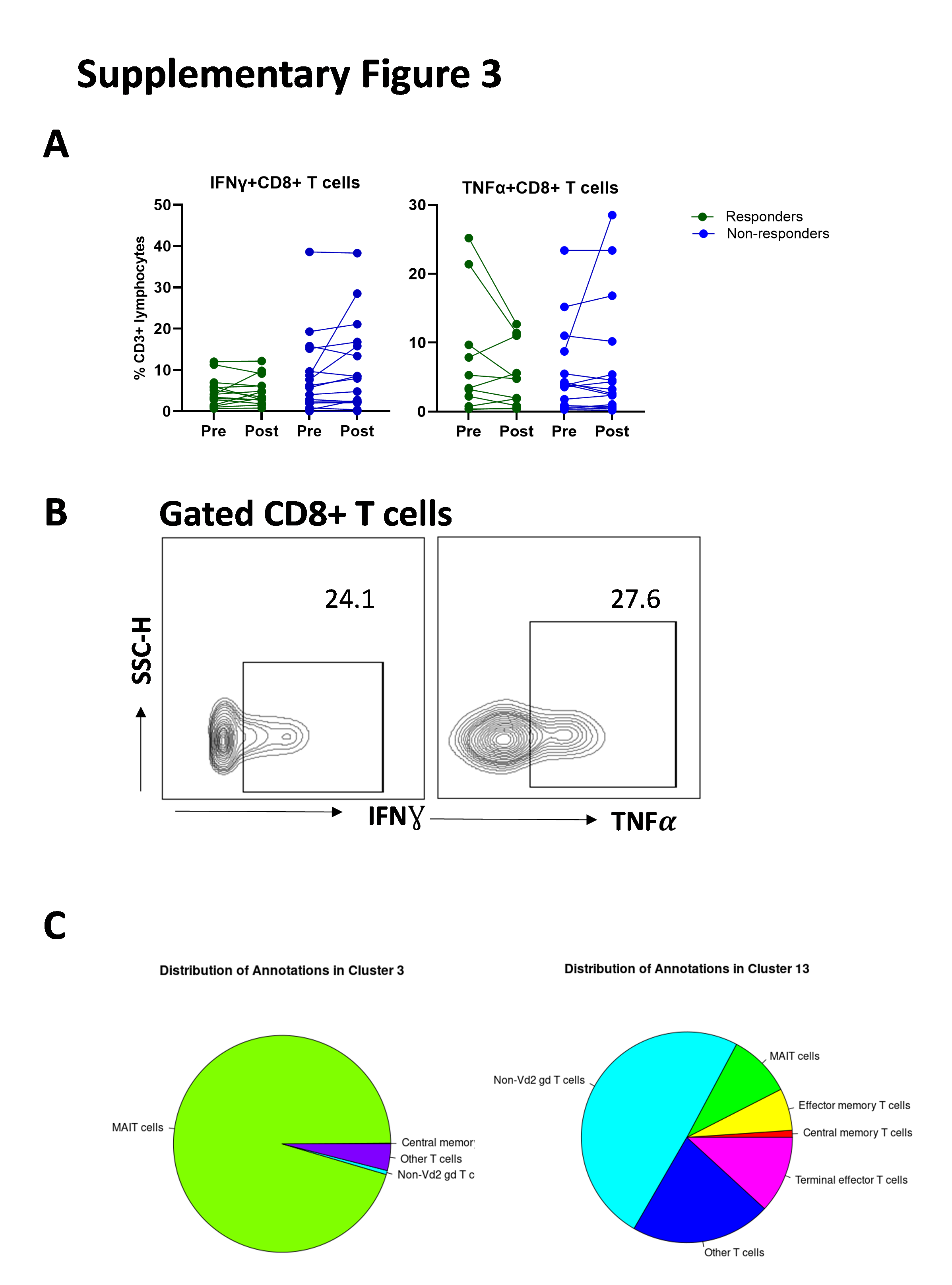

Supplement: Supplementary file 3 — Supplementary file3 (TIF 992 KB) Supplementary figure 3: (A) Ratio pre- versus post-treatment IFNγ and TNFα expression by CD8+ activated effector memory T cells. (B) Flow cytometry plots of IFNγ and TNFα CD8 T cell staining. (C) UMAP gene expression analysis at pretreatment of CD8 T cells reveal clusters dominant at baseline differentiating responders from a nonresponder. [file 262_2025_4086_MOESM3_ESM.tif]
